# Characteristics of *Cyp11a* during Gonad Differentiation of the Olive Flounder *Paralichthys olivaceus*

**DOI:** 10.3390/ijms19092641

**Published:** 2018-09-06

**Authors:** Dongdong Liang, Zhaofei Fan, Yuxia Zou, Xungang Tan, Zhihao Wu, Shuang Jiao, Jun Li, Peijun Zhang, Feng You

**Affiliations:** 1 Key Laboratory of Experimental Marine Biology, National & Local Joint Engineering Laboratory of Ecological Mariculture, Institute of Oceanology, Chinese Academy of Sciences, Qingdao 266071, China; liangdong9531@163.com (D.L.); fanzhaofei0721@163.com (Z.F.); zouyuxia@qdio.ac.cn (Y.Z.); tanx@qdio.ac.cn (X.T.); wzhwzh0123@163.com (Z.W.); jiaoshuang@qdio.ac.cn (S.J.); junli@qdio.ac.cn (J.L.); peijunzhang@qdio.ac.cn (P.Z.); 2 Laboratory for Marine Biology and Biotechnology, Qingdao National Laboratory for Marine Science and Technology, Qingdao 266071, China; 3 College of Earth Sciences, University of Chinese Academy of Sciences, Beijing 10049, China; 4 Center for Ocean Mega-Science, Chinese Academy of Sciences, 7 Nanhai Road, Qingdao 266071, China

**Keywords:** steroidogenic genes, cloning, expression, regulation, flatfish, sex control

## Abstract

The P450 side-chain cleavage enzyme, P450scc (Cyp11a) catalyzes the first enzymatic step for the synthesis of all steroid hormones in fish. To study its roles in gonads of the olive flounder *Paralichthys olivaceus*, an important maricultured fish species, we isolated the *cyp11a* genomic DNA sequence of 1396 bp, which consists of 5 exons and 4 introns. Semi-quantitative reverse transcription polymerase chain reaction (RT-PCR) results indicated that the flounder *cyp11a* was exclusively expressed in gonad and head kidney tissues. Its expression level in the testis was higher than that in the ovary. According to the in situ hybridization patterns, *cyp11a* was mainly expressed in the Leydig cells of the testis, and the thecal cells of the ovary. Immunofluorescence analysis showed that Cyp11a was located in the cytoplasm of the cultured flounder testis cells. Further quantitative real-time PCR results presented the *cyp11a* differential expression patterns during gonad differentiation. Among different sampling points of the 17β-estradiol (E2, 5 ppm) treatment group, *cyp11a* expression levels were relatively high in the differentiating ovary (30 and 40 mm total length, TL), and then significantly decreased in the differentiated ovary (80, 100 and 120 mm TL, *p* < 0.05). The pregnenolone level also dropped in the differentiated ovary. In the high temperature treatment group (HT group, 28 ± 0.5 °C), the *cyp11a* expression level fluctuated remarkably in the differentiating testis (60 mm TL), and then decreased in the differentiated testis (80, 100 mm TL, *p* < 0.05). In the testosterone (T, 5 ppm) treatment group, the *cyp11a* was expressed highly in undifferentiated gonads and the differentiating testis, and then dropped in the differentiated testis. Moreover, the levels of cholesterol and pregnenolone of the differentiating testis in the HT and T groups increased. The expression level of *cyp11a* was significantly down-regulated after the cultured flounder testis cells were treated with 75 and 150 μM cyclic adenosine monophosphate (cAMP), respectively (*p* < 0.05), and significantly up-regulated after treatment with 300 μM cAMP (*p* < 0.05). Both nuclear receptors NR5a2 and NR0b1 could significantly up-regulate the *cyp11a* gene expression in a dosage dependent way in the testis cells detected by cell transfection analysis (*p* < 0.05). The above data provides evidence that *cyp11a* would be involved in the flounder gonad differentiation and development.

## 1. Introduction

Sex hormones play a vital role in fish gonad differentiation and development, and the synthesis process of steroid hormones is very important [1]. As reported in mammals, the P450 side-chain cleavage enzyme, P450scc (Cyp11a) encoded by the *cyp11a* gene is a key enzyme in sex steroid synthesis. By side chain cleavage activity, it can catalyze the conversion of cholesterol to pregnenolone, the initial and rate-limiting reaction in the synthesis process. Then, pregnenolone is further catalyzed by other steroidogenic enzymes such as Cyp17, Hsd3β, Cyp11b1, Hsd11β and Cyp19a to form various sex steroid products [2]. The cloning of the *cyp11a* gene in a few fish has been reported [1,3,4,5,6,7]. Almost similar to those in mammals [1], *cyp11a* in adult fish tissues is mainly expressed in gonadal and kidney tissues [5,8,9]. However, its expression patterns during fish gonad differentiation have not been investigated except for rainbow trout *Oncorhynchus mykiss* [10].

Mechanisms controlling the expression of *cyp11a* have been intensively investigated in mammals. The *cyp11a* transcriptional activity was reported to be regulated by trans-regulators such as members of the orphan nuclear receptor superfamily NR5a2 (previously called SF-1, steroidogenic factor 1) and NR0b1 (previously called DAX-1, dosage-sensitive sex reversal, adrenal hypoplasia congenital critical region, on chromosome X, gene 1) [11,12,13]. Dax-1 is expressed in the same cell types as SF-1, and interacts directly with SF-1 to repress steroidogenic gene expression [14,15]. SF-1 can stimulate basal transcription of *cyp11a1* in either adrenal or kidney-derived nonsteroidogenic cell lines [11]. The cyclic adenosine monophosphate (cAMP) could also affect transcription of *cyp11a1* through its TATA binding site [16]. However, few studies on the gene regulation in fish have been reported, except for the role of GTH in brown hagfish *Paramyxine atami* [7] and an impact of exogenous fadrozole (model aromatase inhibitor) in the fathead minnow *Pimephales promelas* [17].

The olive flounder *Paralichthys olivaceus* is one of the most economically important marine aquaculture species in China, Japan and Korea. There are growth differences between female and male flounders [18]. Therefore, studies on flounder sex control and its molecular regulation are valuable. So far, no sex chromosome or sex determination gene has been found although its genomic data have been published [19]. The flounder sex has been proved to be determined by genotype, while environmental factors could also alter the gender [20]. Our previous study showed that 17β-estradiol (E2) in juvenile flounder plays a major role in the process of ovary differentiation, and temperature-induced masculinization was suggested to be mainly due to a decrease of E2 concentration during this process [21,22]. As mentioned above, the sex hormone synthesis-related gene, *cyp11a*, may be indispensable to gonad differentiation and development in the flounder. In addition, *cyp11a* expression was also found to be sexually dimorphic based on transcriptomics in the flounder testes and ovaries [23]. However, little information is known about the function of flounder *cyp11a*.

In the present study, we analyzed *cyp11a* expression in adult flounder gonads and during gonad differentiation as well as the relevant hormone levels. The effects of cAMP, NR5a2 and NR0b1 on its expression were also investigated. We provide new insights into the study of flounder steroidogenesis and gonad differentiation.

## 2. Results

### 2.1. Flounder cyp11a Gene and Its Phylogenetic Tree

**Figure 1:**
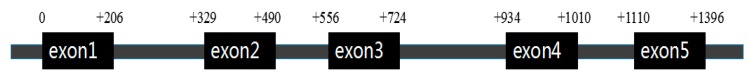
Genomic structure of the flounder *cyp11a* gene. Shaded boxes indicate exons, and introns are between them.

The genomic sequence of the flounder *cyp11a* we obtained was 1396 bp, which contains 5 exons and 4 introns, and its cDNA sequence was 828 bp (Figure 1). The cloned flounder Cyp11a amino acids share 76.7% with *T. rubripes*, 77.1% with *Ory. latipes*, 69.4% with *D. rerio*, 75.9% with *M. zebra*, 75.6% with *Ore. niloticus*, 75.0% with *S. salar*, and 75.0% with *On. mykiss*.

**Figure 2:**
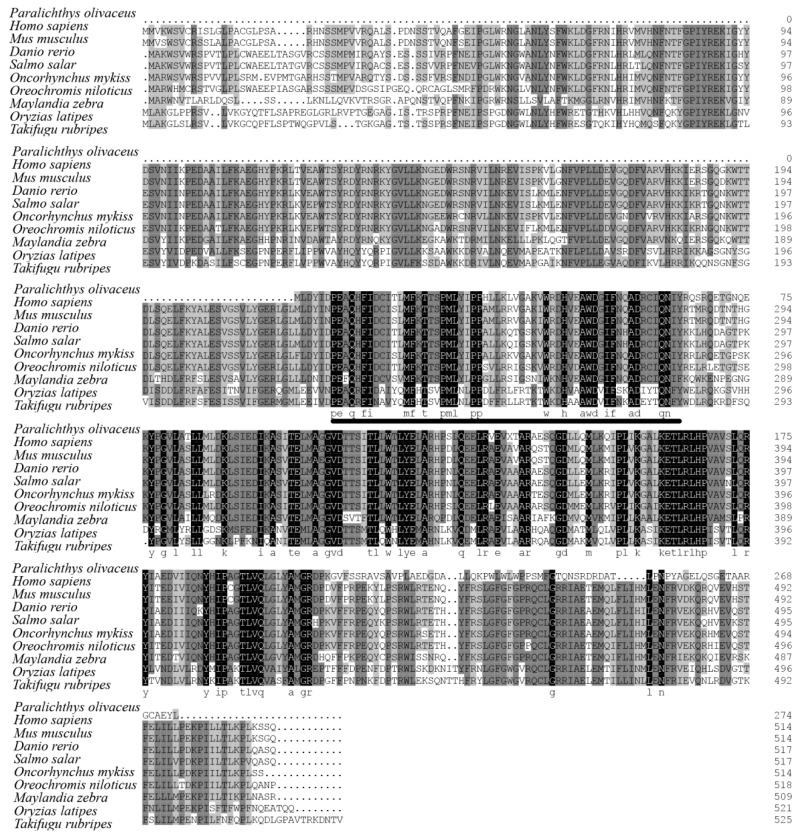
Sequences alignments of Cyp11a. The black background letters indicate the conserved amino acid residues. The putative conserved P450scc superfamily domains including the steroid-binding domain and the heme-binding region are underlined.

**Figure 3:**
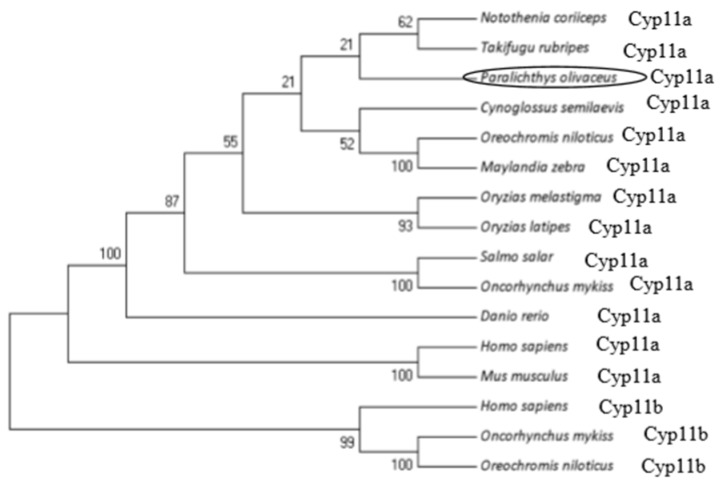
Phylogenetic tree of vertebrate Cyp11a and Cyp11b proteins. Values at the branch points indicate the percentage of 1000 bootstrap replicates supporting the division. The circled words is scientific name of the flounder.

Multiple alignments indicated that the flounder Cyp11a protein sequence contains the putative conserved P450scc superfamily domains including the steroid-binding domain and the heme-binding region (Figure 2). Phylogenetic tree analysis showed that flounder Cyp11a was clustered with Cyp11a proteins from other fish such as *N. coriiceps* and *T. rubripes* except for Cyp11b (Figure 3)*.* These above results could imply that the cloned gene should be *cyp11a*.

### 2.2. Tissue Distribution of Flounder cyp11a

**Figure 4:**
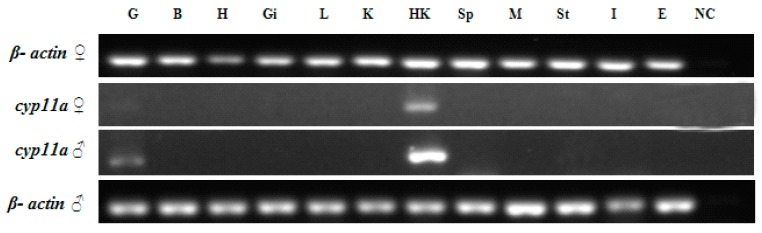
The tissue distribution of *cyp11a* gene detected with RT-PCR. G, testis/ovary; B, brain; H, heart; Gi, gill; L, liver; K, kidney; HK, head kidney; Sp, spleen; M, muscle; St, stomach; I, Intestines; E, eye; C, negative control.

According to the semi-quantitative reverse transcription polymerase chain reaction (RT-PCR) results in adult tissues, flounder *cyp11a* was expressed in gonads, and the expression level was higher in the testis than in the ovary (Figure 4). The gene was also strongly expressed in the head kidney (HK) of both sexes.

### 2.3. Spatial Expression Analysis of Flounder cyp11a Gene in Gonads 

**Figure 5:**
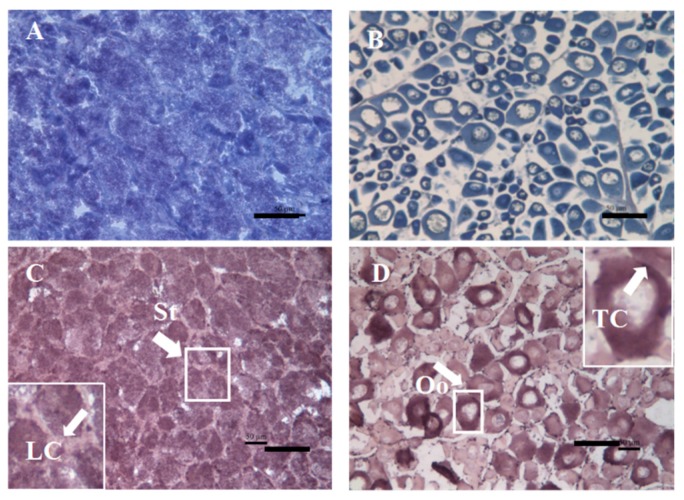
Expression of *cyp11a* in the flounder testis and ovary by in situ hybridization (ISH) analysis**.** Cyp11a mRNA was expressed in the Leydig cells (LC) and spermatid (St) of the testis, and in the thecal cells (TC) of the ovary. Oo, oocytes. (**A**), testis by hematoxylin/eosin (HE) staining; (**B**), ovary by HE staining; (**C**), testis by ISH; (**D**), ovary by ISH. Arrowheads indicate positive signals of *cyp11a*. Scale bar, 50 μm.

The spatial expression of *cyp11a* in flounder gonads was determined by in situ hybridization (ISH). After morphological observation and histological analysis, the testis and ovary of the wild-type adult flounder samples were confirmed to be at stage II and stage III, respectively (Figure 5A,B). Compared with the results of HE staining slides, flounder *cyp11a* mRNA was expressed only in the Leydig cells (LC) and spermatid (St) of the testis (Figure 5C), and in the thecal cells (TC) of the ovary (Figure 5D).

**Figure 6:**
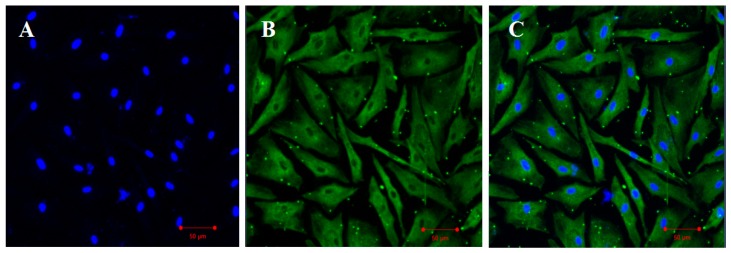
Expression of Cyp11a protein in the cells of the flounder testis cell line by immunofluorescence (IF). Cyp11a protein was located in the cytoplasm of the testis cells (green signal). The blue signal indicates cell nucleus by 4′,6-diamidino-2-phenylindole (DAPI) staining. (**A**), DAPI; (**B**), IF results; (**C**), Merge. Scale bar, 50 μm.

The expression position of Cyp11a protein in the flounder testis cells was analyzed by immunofluorescence (Figure 6). It was expressed in the cytoplasm of the testis cells (Figure 6B,C).

### 2.4. Analyses of cyp11a Expression, and Cholesterol and Pregnenolone Levels during Flounder Gonad Differentiation

#### 2.4.1. Expression of Flounder *cyp11a*

**Figure 7:**
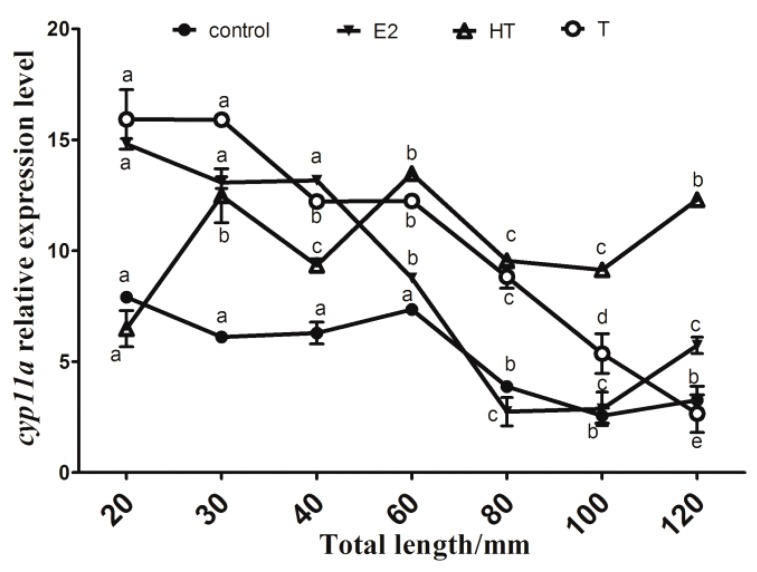
Flounder *cyp11a* gene expression levels in gonad during gonad differentiation period. Data are means ± standard error of the mean (S.E.M.) of three independent samples. Different lowercase letters (a, b, c, d and e) indicate a significant difference in values among sampling points in each group (*p* < 0.05). Control, control group; E2, estradiol treatment group; HT, high temperature treatment group; T, testosterone treatment group.

Histological observation revealed that the sex ratios (percentages of female) in the control, estradiol (E2), high temperature (HT) and testosterone (T) treated groups were 75%, 100%, 0% and 0%, respectively (*n* = 20), when the juveniles reached 150 mm total length (TL) [24]. According to our previous study [25], the flounder ovary differentiation started when ovarian cavity was firstly observed at 38.0 ± 1.7 mm TL, and oocytes were subsequently detected at 86.5 ± 5.9 mm TL. Testis differentiation was initially observed at 63.5 ± 3.4 mm TL. The seminal lobule appeared at approximately 76.0 mm TL. Expression levels of *cyp11a* during the gonad differentiation period shown by quantitative PCR (qPCR) were more elevated until the juveniles reach 60 mm TL and significantly dropped afterwards in the control group without treatment (*p* < 0.05) (Figure 7). The expression level of *cyp11a* in the E2 group was significantly more elevated at 20, 30 and 40 mm TL, then significantly decreased at 60, 80, 100 and 120 mm TL (*p* < 0.05), while in the T group its expression levels remained relatively higher before and at 60 mm TL, and then dropped at 80, 100 and 120 mm TL. In the HT group, *cyp11a* expression levels showed similar patterns to those in the T group during testis differentiation period, reaching peak at 60 mm TL and decreasing at 80 and 100 mm TL (*p* < 0.05).

#### 2.4.2. Cholesterol and Pregnenolone Levels

**Figure 8:**
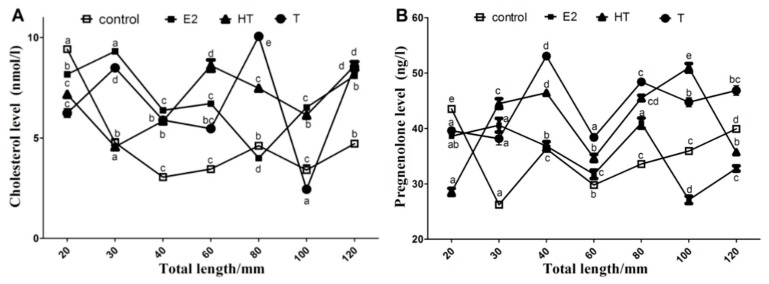
Cholesterol (**A**) and pregnenolone levels (**B**) in the flounder gonad during gonad differentiation period. Data are means ± S.E.M. of three independent samples. Different lowercase letters (a, b, c, d and e) indicate a significant difference in values among sampling points in each group (*p* < 0.05). Control, control group; E2, estradiol treatment group; HT, high temperature treatment group; T, testosterone treatment group.

In the control group, the cholesterol level significantly dropped during ovary differentiation period (30, 40 and 60 mm TL) (*p* < 0.05), and remained stable in the differentiated gonads (≥80 mm TL) (Figure 8). Pregnenolone level was higher at 20 mm TL, dropped at 30 mm TL (*p* < 0.05), and increased at 40, 60, 80, 100 and 120 mm TL (*p* < 0.05). In the E2 group, the cholesterol level increased at 30 mm TL, dropped at 40, 60, 80mm TL, then increased at 100 and 120 mm TL. The pregnenolone level increased at 30 mm TL, dropped at 40 and 60 mm TL, and then increased. In the T and HT groups, the cholesterol and pregnenolone levels all increased during the testis differentiation period (60 and 80 mm TL).

### 2.5. Regulators’ Effect on Flounder cyp11a Expression

#### 2.5.1. Impact of cAMP on Flounder *cyp11a* Expression in the Testis Cells 

**Figure 9:**
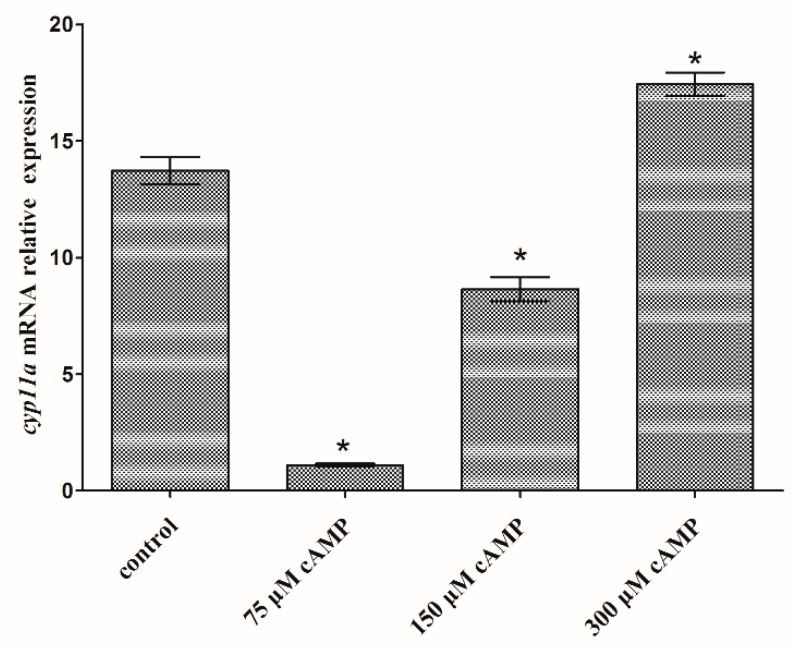
Flounder *cyp11a* gene expression levels in the flounder testis cells after treated with 0, 75, 150 and 300 μM cAMP. Data are presented as mean ± S.E.M. of three independent samples. Asterisk indicates a significant difference from the control (*p* < 0.05).

The expression levels of *cyp11a* were significantly down-regulated in the testis cells treated with 75 and 150 μM cAMP, and up-regulated in the testis cells treated with 300 μM cAMP (*p* < 0.05) (Figure 9).

#### 2.5.2. NR5a2 and NR0b1 Regulating Expression Level of *cyp11a*

**Figure 10:**
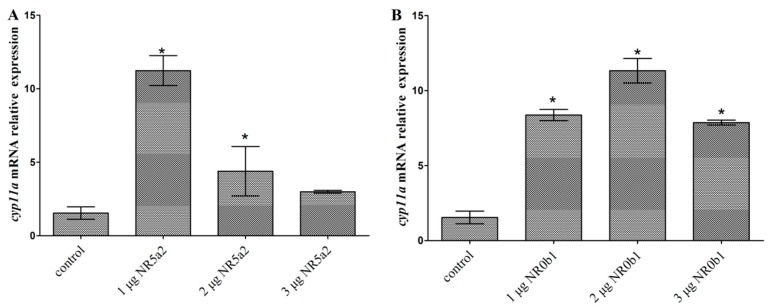
NR5a2 (**A**) and NR0b1 (**B**) regulating the expression of *cyp11a* in the flounder testis cells. Data are means ± S.E.M. of three independent samples. Asterisk indicates a significant difference from the control (*p* < 0.05).

Doses of 1 and 2 μg NR5a2 significantly up-regulated the expression level of *cyp11a* (*p* < 0.05), while 3 μg NR5a2 did not (Figure 10A)*.* All 1, 2 and 3 μg NR0b1 could significantly up-regulate the expression level of *cyp11a* in the testis cells (*p* < 0.05) (Figure 10B).

## 3. Discussion

### 3.1. Characteristics of the Flounder cyp11a

Cyp11a (P450scc) belongs to the cytochrome P450 family (CYP). CYP11A1 catalyzes cholesterol to pregnenolone, a sole precursor of all other steroids, with the oxidative side chain cleavage reaction [5]. We cloned flounder *cyp11a* cDNA and its genomic DNA sequences, and found that the deduced Cyp11a amino acid sequence has P450 superfamily domains including the steroid-binding domain and the heme-binding region which are conserved in teleosts [9]. Parajes et al. [26] cloned two *cyp11a* genes (*cyp11a1* and *cyp11a2*) in zebrafish and reported that *cyp11a2* shares 85% identity with *cyp11a1* indicating this gene’s conservation. However, similar to catfish *Clarias batrachus* [9], only one transcript was found in the flounder.

Localization of *cyp11a* mRNA previously reported in fish was mainly expressed in gonads and head kidney [5,8]. Flounder *cyp11a* was also expressed in the gonad and head kidney tissues, and there was sexually dimorphic expression with higher level in the testis than in the ovary. Further ISH results showed that *cyp11a* was expressed in the Leydig cells of testis and thecal cells of ovary, as was detected in brown hagfish and rainbow trout [7,27]. These cells are all the typical steroid producing cells of gonad, and *cyp11a*’s tissue distribution in fish is in accordance with its function in the steroidogenesis [7].

### 3.2. Differential Patterns of cyp11a Expression, and Cholesterol and Pregnenolone Levels during the Flounder Gonad Differentiation

Cyp11a is a fundamental enzyme in the steroidogenic pathway in teleost species [1] and plays roles in gonad development as shown in adult zebrafish [28] and brown hagfish [7]. Various steroids and xenochemicals could alter *cyp11a* gene expression. Androgen and estrogen decreased the level of *cyp11a* mRNA in rainbow trout pre- and post-differentiating testis [10,29,30], while, androgen strongly increased the mRNA level of *cyp11a* in the rainbow trout’s pre-differentiating ovary [10]*.* However, in the yellow catfish, *Pelteobagrus fulvidraco*, *cyp11a* expression in the ovary was reduced after 17a-methyltestosterone (MT) treatment and increased upon 17a-ethinylestradiol (EE2) treatment [31]. Water temperature, an important environmental factor for fish, has been proved to control fish gonad differentiation, whose effects on related genes’ expression are very important to explain possible sex ratio shift and other effects on sex differentiation [32]. There are few studies on *cyp11a* expression patterns under high temperature and exogenous hormones treatment during gonad differentiation. The present study showed that flounder *cyp11a* expression was significantly reduced in the differentiating ovary of the control and E2 groups (*p* < 0.05) as observed in maturing zebrafish follicles [28]. On the contrary, in the HT and T groups, *cyp11a* expression was higher before flounder testis differentiation, but dropped during the differentiation [25] as observed in the pejerry *Odontesthes bonariensis* [33], which could reinforce the fact that gonadal steroidogenesis in teleosts is active before histological differentiation, while in the brown hagfish, *cyp11a* expression increased with testis development [7]. The reason for the differences in these species was not clear, and different fish species might have different expression patterns. Further relevant studies in more fish species should be performed.

Cholesterol is an important factor that regulates the activity of P450scc [8]. In this study we analyzed the levels of cholesterol and its conversion product, pregnenolone, during gonad differentiation period. In the E2 groups, changes of cholesterol and pregnenolone levels during and after ovary differentiation were consistent with the expression levels of *cyp11a*. Sex steroids may be unnecessary after ovary differentiation, so this rate-limiting reaction in the synthesis of steroid hormones is repressed. On the other hand, the rise of cholesterol and pregnenolone levels during the male gonad differentiation period in the T and HT treatment groups meant that more steroids were synthesized, which indicates high demands of steroids in the process. Positive and negative correlations between endogenous 11-keto testosterone (11-KT) and the expression of testis-biased or ovary-biased genes by MT treatment, and endogenous E2 and gene expression by EE2 treatment were reported in yellow catfish [31]. Our previous result [32] indicated that high temperature and exogenous T could repress *cyp19a* expression to promote testis formation with higher endogenous 11-KT and T levels, while exogenous E2 could up-regulate *cyp19a* expression to produce E2 and induce ovary formation. Together with the present study, overall results indicate that there may be a negative feedback on regulation of the *cyp11a* gene expression through hypothalamus-pituitary-gonadal (HPG) axis during testis differentiation. The up-regulation of *cyp11a* might contribute to masculinization under HT and T treatment.

### 3.3. The Function of cAMP and Transcription Factors on Flounder cyp11a

The study on *cyp11a* promoter activity regulation includes cis-regulatory elements and trans-regulators. The cAMP-responsive sequence in mammal *cyp11a* promoter is important to hormonal-dependent stimulation of *cyp11a* transcription via the cAMP signaling pathways [28]. *Cyp11a* promoter includes a TATA box at −24/−29 which may be bound by factors that contribute to cAMP response depending on the promoter context. Replacing the TATA binding site abolishes its response to cAMP [16]. Down- and up-regulation of *cyp11a* expression by cAMP administration indicates that flounder *cyp11a* expression could also be regulated by cAMP and the regulation was dosage dependent. NR5a2 (i.e., SF-1) and NR0b1 (i.e., Dax-1) are the mainly regulated factors in the steroidogenesis [34]. These two trans-regulators could regulate *cyp11a* transcriptional activities through their specific cis-elements or protein–protein interactions [11]. NR5a2 plays an important role in the expression of tissue-specific genes and regulates the expression of steroidogenic genes, such as *cyp11a* [11]. It is mainly expressed in the testis, ovary and adrenal cortex, controlling the tissue-specific transcription of *cyp11a* in these tissues [34]. The binding site (5′-CAAGGCC-3′) of NR5a2 was found in the promoter of flounder *cyp11a* by analyzing its genome sequence [19]. The present results showed that NR5a2 significantly up-regulated the expression of the flounder *cyp11a* in the testis cells in a dosage-dependent way (*p* < 0.05), and high concentration could repress the up-regulated gene expression. NR0b1 is expressed in the same cell types as NR5a [35]. It interacts directly with NR5a to repress steroidogenic gene expression [14,15,36]. Overexpression of the flounder NR0b1 in the testis cells could also significantly up-regulate *cyp11a* expression (*p* < 0.05), but the activating function decreased under the higher NR0b1 concentration. More factors could regulate the expression of *cyp11a*. Therefore, more regulation factors in the flounder should be studied to obtain detailed information on *cyp11a* function.

In conclusion, this study indicates that flounder *cyp11a* expression is sexually dimorphic. HT, T and E2 could affect the expression levels of *cyp11a* and the synthesis of pregnenolone during flounder gonad differentiation. Furthermore, cAMP could significantly regulate the *cyp11a* gene expression in the testis cells in a dose-dependent way. Transcription factors (NR5a2 and NR0b1) also significantly up- or down-regulated the expression level of *cyp11a*, and the effects were also dose-dependent. The above results suggest that *cyp11a* may be involved in flounder gonad differentiation and development, and its expression is regulated by cAMP, NR5a2 and NR0b1. How *cyp11a* functions and how it is regulated in these processes remain the focus of further study.

## 4. Materials and Methods

### 4.1. Ethics Statement

The study was conducted in accordance with the guidelines and regulations established by the Chinese Government Principles for the Utilization and Care of Animals Used in Testing, Research, and Training. All other applicable international, national, and institutional guidelines for the care and use of animals were followed by the authors. All the experimental flounder work and protocols were permitted and approved by the Institute of Oceanology, Chinese Academy of Sciences (41276171 approved on 17 Aug. 2017 and 31772834 approved on 17 Aug. 2011).

### 4.2. Fish and Samples Collection

The wild-type adult flounders (300 ± 20 mm TL) were cultured at the institute aquarium [24]. Twelve tissues (ovary/testis, brain, heart, muscle, head kidney, kidney, intestine, spleen, liver, stomach, gill and eye) were, respectively, dissected from three male and three female flounders after anesthetization with tricaine methane sulfonate (MS-222). Gender and development stage of gonads were confirmed by frozen sections of gonads stained with hematoxylin/eosin (HE). Parts of the testes or ovaries were fixed at 4 °C with 4% paraformaldehyde (PFA) in 1× phosphate buffered saline (PBS, pH 7.4) for ISH analysis, and the rest and other tissue samples were stored immediately in liquid nitrogen for RNA isolation. 

All genetically-female flounders were produced with artificially induced meio-gynogenesis as described previously [37]. Healthy all-female flounder juveniles were divided into four groups of 250 individuals each (HT, T, E2 treatments and a control) and reared in 90 L plastic tanks. The treatment period was from 15 mm TL to 120 mm TL which is the sensitive period of the flounder gonad differentiation [25]. The methods to raise water temperature in the HT group and to make 5 ppm T or 5 ppm E2 diets, sampling scheme, and identification of phenotypic sex were described in the previous study [24]. The rearing temperature was maintained at 28 ± 0.5 °C in the HT group and at 20–22 °C in the other groups. Then, all juveniles (>120 mm TL) in each experimental group were cultured under the same condition as the control group (at 20–22 °C and fed with commercial diets). The experiment was duplicated.

### 4.3. Total RNA Extraction, cDNA Synthesis and Genomic DNA Extraction

Total RNA was isolated from the tissue and gonadal samples by using Trizol reagent (Invitrogen, Carlsbad, CA, USA) following the manufacture’s protocol. The quantity and purity of the RNA were assessed by electrophoresis in 1% agarose gel and Nanodrop2000 (Thermo Scientific, Waltham, MA, USA). Around 2 μg RNA was used in the first-strand cDNA synthesis with the EasyScript^®^ one-step gDNA removal and cDNA synthesis superMix Kit (Transgen, Beijing, China). The cDNA was preserved at −20 °C. Genomic DNA (gDNA) was isolated from the gonadal tissues using the marine biology tissue gDNA extraction kit (Tiangen, Beijing, China).

### 4.4. *Cyp11a* Cloning

The *cyp11a* sequence based on the flounder testis and ovary transcriptome data [23] was cloned and verified. Primers (*cyp11a*-oF: ATGCTGGACTACATTGACCCTG, *cyp11a*-oR: GAATTCAAGGTACTCTGCACA) were designed using Primer Premier 6.0 (Premier Biosoft, Palo Alto, CA, USA). The polymerase chain reaction (PCR) for cDNA cloning was similar to that for genomic sequence, containing 1 μL of cDNA from testis (or gDNA) (50 ng/μL), 10 μL of 2× GoldStar MasterMix (CWBIO, Beijing, China), 1 μL of forward primer (10 mM), 1 μL of reverse primer (10 mM) and 7 μL of RNase-free water. The PCR was performed as follows: initial denaturation at 95 °C for 10 min, 35 cycles of 95 °C for 30 s, 56 °C for 30 s, and 72 °C for 50 s, and a final extension at 72 °C for 10 min. All the PCR products were electrophoresed with 1% agarose and purified using E.Z.N.A.^®^ Gel Extraction Kit (Omega Bio-Tek, Norcross, GA, USA). The target fragments were cloned into pEASY-T3 vector (Transgen) and sequenced.

### 4.5. Phylogenetic Tree Construction and Multiple Alignments

The phylogenetic tree of Cyp11a and Cyp11b was established using the neighbor-joining method implemented with MEGA5.1 software (Arizona State University, Tempe, AZ, USA) [38]. Values at the branch points indicate the percentage of 1000 bootstrap replicates supporting the division. Multiple alignments of deduced amino acid sequences were performed based on Cyp11a amino acid sequences of several species using BioEdit7.0 [39]. The Cyp11a sequences were selected from GenBank or Ensemble, and the animal scientific names and accession nos. are: *Homo sapiens* (M14565.1), *Mus musculus* (NM_019779.4), *Notothenia coriiceps* (XM_010795923.1), *Salmo salar* (XP_013981585.1), *Oryzias melastigma* (JX454604.1), *Oreochromis niloticus* (XM_003440441.3), *Takifugu rubripes* (XM_003967446.1), *Danio rerio* (AF527755.1), *Maylandia zebra* (XM_004553101.2), *Oncorhynchus mykiss* (S57305.1), *Oryzias latipes* (EF537029.1) and *Cynoglossus semilaevis* (XM_008312076.2). The *Cyp11b* sequences were selected from GenBank or Ensemble, and the animal scientific names and accession nos. are: *H. Sapiens* (NC_000008.11), *On. mykiss* (NC_035078.1) and *Ore. niloticus* (NC_031976.1).

### 4.6. Semi-Quantitative Reverse Transcription Polymerase Chain Reaction (RT-PCR)

Tissue samples from three male and three female wild-type adult flounders were analyzed to evaluate the expression of the *cyp11a* by using RT-PCR. The primers (*cyp11a*-qF: TGGACGTTGTACGAACTGGC, *cyp11a-*qR: GGGACGGCTGATACTGCTCT) were designed using Primer Premier 6.0 based on the flounder *cyp11a* cDNA sequence. The *β-actin* (*β-actin*-F: ACTACCTCATGAAGATCCTG, *β-actin*-R: TTGCTGATCCACATCTGCTG) was selected as the internal reference gene. PCR reaction mixture was exactly the same as described in the Section 4.4. PCR was performed as follows: 95 °C for 10 min, and 30 cycles of 95 °C for 30 s, 55 °C for 30 s, and 72 °C for 20 s, followed by a final extension of 72 °C for 10 min.

### 4.7. In Situ Hybridization (ISH) and Immunofluorescence Analyses

A frozen section of wild-type adult flounder gonads was made for histological analysis as control [24]. A plasmid *cyp11a*-pEASY-T3 was constructed and confirmed by sequencing. Antisense digoxigenin-labeled RNA probes were produced by transcription from the linearized *cyp11a*-pEASY-T3 using a RNA labeling kit (Roche, Germany). ISH was performed as described by Kobayashi et al. [40].

Flounder testis cells were biopsied and cultured according to the method described previously [41]. Single cell could be observed under microscope after the testis cells were cultured for 6 passages. When the testis cell numbers reached 2.5 × 10^5^, cells were used for immunofluorescence (IF) by using the *Cyp11a* primary rabbit antibody (ABclonal, Woburn, MA, USA). The dilutions of primary antibody was 1:100, and the second antibody was 2 μg/mL goat anti-rabbit IgG (H + L) (Alexa Fluor^®^ 488) (Dilutions = 1:1000, Abcam, Cambridge, UK). The pictures were taken under the confocal laser scanning microscopy LSM710 (Zeiss, Germany).

### 4.8. The Testis Cells Stimulated with Cyclic Adenosine Monophosphate (cAMP)

In order to study the role of cAMP on *cyp11a* expression, we treated the flounder testis cells with cAMP. When the number of the cultured testis cells reached 4.0 × 10^5^, cells were inoculated into the 6 well-plates (NEST, Palo Alto, CA, USA) and treated with 0, 75, 150 and 300 μM cAMP (Solarbio, Beijing, China) [42], respectively. All the experiments were repeated three times for biological replicates (i.e., *n* = 3). After 72 h in culture, the testis cells were harvested for RNA isolation, and *cyp11a* expression levels were analyzed by quantitative real-time PCR (qPCR).

### 4.9. Cell Transfection Assay

We overexpressed the NR5a2 and NR0b1 in the flounder testis cells by cell transfection to examine their regulation roles in the *cyp11a* expression. The NR5a2 and NR0b1 expression plasmids were constructed by ligating their open reading frame (ORF) into pcDNA3.1 (Invitrogen, Carlsbad, CA, USA) as described by Liang et al. [24], respectively. When the cultured testis cell number reached 4.5 × 10^5^, cells were inoculated into the 6 well-plates (NEST) and respectively transfected with 0, 1, 2 and 3 μg pcDNA3.1-*NR5a2* or pcDNA3.1-*NR0b1* plasmids following the protocol of Lipofectamine 2000 reagent (Invitrogen). All the experiments were repeated three times for biological replicates (*n* = 3). After 48 h in culture, the testis cells were harvested for RNA isolation, and *cyp11a* expression levels were analyzed by using qPCR.

### 4.10. Quantitative PCR (qPCR)

All the qPCRs were conducted on an Applied Biosystems QuantStudio 6 qPCR machine (Life technologies, Carlsbad, CA, USA) in a 20 μL volume containing 5 μL of 2× TransStart Top Green qPCR SuperMix (Transgen), 0.4 μL of each forward and reverse primer (10 μM), and 1 μL of cDNA (50 ng/μL). Melting curves were used to confirm the specificity of each primer and showed no primer-dimers present. Primers (qRT-*cyp11a*-F: TGGGACGGGATCTTCAACC; qRT-*cyp11a*-R: CACAGCAGCGTTATAGAAGTCGT) based on cDNA sequence of flounder *cyp11a* were designed using Primer Premier 6.0. The *β-actin* was selected as the internal reference gene according to Zheng and Sun [43]. The qPCR condition was 94 °C for 30 s, and 40 cycles of 94 °C for 5 s, 55 °C for 20 s, and 72 °C for 20 s, followed by measuring melting curves. Samples were run in triplicates and relative gene expression levels were calculated by the 2^−ΔΔ*C*t^ method [44].

### 4.11. Enzyme-Linked Immunosorbent Assay (ELISA)

Hormones were extracted from juvenile flounder according to the methods used by Fan et al. [32]. Briefly, after stomach and intestine were removed, the juveniles were homogenized in 1 mL/g 50% ethanol. The supernatants with dissolved hormones were collected after centrifugation of homogenates at 6000 rpm, 4 °C, for 10 min. The hormones were further extracted twice with dichloromethane, and then were dissolved in 0.5 mL 1× PBS (pH 7.4, containing 1% BSA) after evaporation of dichloromethane. The concentrations of cholesterol and pregnenolone were detected by using enzyme-linked immunosorbent assay (ELISA) kits (XF Biotech, Shanghai, China). Seven samples in each group were measured, and all assays were repeated three times.

### 4.12. Statistical Analysis

All data were shown as mean ± standard error of the mean (S.E.M.). One-way analysis of variance (ANOVA) followed by the Dunnett test or Tukey multiple comparison test (for comparing all pairs of groups) in the SPSS software package (IBM SPSS, Armonk, NY, USA) was used to test differences in gene expression levels among groups. Two-tail Student’s *t*-tests were used to determine the difference between means of pairs. Significance was set at *p* < 0.05.
